# Letter from Messrs. William Wood & Company

**Published:** 1897-05

**Authors:** 

**Affiliations:** New York


LETTER FROM MESSRS. WILLIAM WOOD & COMPANY.

NEW YORK, April 1, 1897.

*To the Editor :*

Sir—We notice that in your issue for April our letter of March 11th is headed "Reply of Messrs. William Wood & Co."

Our letter of that date is not a reply to Dr. Gould's communication, as can be seen by reading it, or to any of his letters ; nor would we reply to them.

Also the opening line, which reads, "Referring to the foregoing communication of Dr. Gould, we beg to state that," etc., is not ours, but has been changed by someone in your office.[1]

Our letter was an independent communication, explaining our course to the medical profession, and intended to be printed as such ; not as a "*reply*" to anyone.

Sincerely yours,

WILLIAM WOOD & COMPANY.

---

1. The letter of Messrs. William Wood & Co., referred to, was addressed personally to the undersigned and not to the JOURNAL. As that was a bar to its publication we asked Messrs. Wood if they desired it published. They replied affirmatively, whereupon we changed the address and the opening sentence, to make it conform to the usual style of correspondence to appear in print, which in nowise affected its purport or meaning. As to the criticism regarding the heading, if it is not a "reply" to Dr. Gould we are at a loss to know what its object is. We might have headed it "Our New York letter," but we think the heading chosen was nearer correct. We exercised only our editorial prerogative in the premises and now publish the foregoing letters as a conclusion to the whole matter in so far as the JOURNAL is concerned.--WILLIAM WARREN POTTER.

